# A simple CT score predicts early neurological disability and survival in supratentorial intracerebral hemorrhage - The intracerebral mass and brain edema score (IMBES)

**DOI:** 10.1016/j.bas.2025.104226

**Published:** 2025-02-28

**Authors:** Ralf Watzlawick, Panagiotis Fistouris, Ahmed Elbaz, Pierre Scheffler, Alix Bührle, Marc Hohenhaus, Ramy Amirah, Roland Roelz, Mukesch Shah, Oliver Schnell, Samer Elsheikh, Christian Taschner, Christian Fung, Jürgen Beck

**Affiliations:** a Department of Neurosurgery, Medical Center - University of Freiburg, Faculty of Medicine, University of Freiburg, Freiburg, Germany; b Department of Neuroradiology, Medical Center - University of Freiburg, Faculty of Medicine, University of Freiburg, Freiburg, Germany

**Keywords:** Intracerebral hemorrhage, Neurological outcome, Radiological score, Stroke

## Abstract

**Introduction:**

Treatment of spontaneous intracerebral hemorrhage (ICH) remains challenging, and intracerebral mass and brain edema (IMBE) are accused of being the main factors influencing patient course.

**Research question:**

CT scan assessment of the space-occupying effect after initial ICH was evaluated using an IMBE-score to detect the sulcal effacement of the subarachnoid space.

**Material and methods:**

Supratentorial ICH-patients within a 10 years observation period were identified. Two independent reviewers screened each CT scan in three defined axial planes. Where the combined mass effect of hemorrhage and edema showed sulcal effacement of more than half of the hemisphere, one point was assigned, resulting in an IMBES between 0 and 6. The primary endpoint was neurological outcome measured by the modified Rankin Scale (mRS) and mortality.

**Results:**

We identified 762 patients, median age was 75.4 years (IQR: 64.3–81.1) and mean ICH volume was 46.1 cc. Multiple regression for mRS at discharge (mean: 12.5 days, IQR: 7.1–22.3) identified age, presence of intraventricular hemorrhage, ICH volume, renal insufficiency, intake of anticoagulants and IMBES as statistically significant variables. This was confirmed during follow-up examination, although ICH volume was not significantly associated with neurological outcome.

**Discussion and conclusion:**

We observed a decreased neurological recovery and an increased mortality for patients with high IMBES during acute care and at early follow-up, indicating that IMBES had the strongest association in all regression analysis. We conclude that the fast and simple IMBES may be a useful tool to estimate patient risk for impaired neurological outcome and death.

## Highlights

• This retrospective study identified 762 patients with spontaneous intracerebral hemorrhage.
• The cumulative space-occupying effect of ICH and edema is assessed by IMBES on three axial CT scans.
• This simple radiological assessment can be quickly performed using routine non-contrast CT scans.
• The IMBES was a significant predictor of neurological outcome and mortality.
• Based on sulcal effacement the IMBES outperforms ICH volume in predicting neurological outcome.

## Abbreviations

CI: Confidence intervals
CT: Computed tomography scan
EVD: External ventricular drainage
ICH: Intracerebral hemorrhage
IMBES: Intracerebral Mass and Brain Edema Score
IQR: Interquartile range
IVH: Intraventricular hemorrhage
mRS: Modified Rankin Scale
n: Number of patients
cc: Cubic centimeter [cm^3^]

## 1 Introduction

Intracerebral hemorrhage (ICH) remains one of the most severe forms of stroke accompanied by high mortality and poor long-term functional outcome (Qureshi et al., 2009; Collaborators, 2021). Neurological compromise caused by the ICH is a sequelae of location, extension, hematoma volume, toxic mediators and perihematomal edema. Primary brain damage resulting from the immediate destruction of brain tissue by ICH must be distinguished from secondary brain damage (Lok et al., 2011). One pathomechanism of secondary brain damage is the space-occupying effect resulting in pathologically increased intracranial and perihemorrhagic pressure. The space-occupying effect is exerted by the sum of ICH volume and perihematomal edema volume (Murthy et al., 2015).

The ICH volume can be measured volumetrically using computer-based algorithms or clinically using some heuristics. The perihematomal edema volume is even more complex to acquire. In addition, a ballooned ventricular system from intraventricular hematoma extension and/or hydrocephalus contributes to this effect and complicates the radiological assessment (Hanley, 2009). These factors affect a variable amount of brain tissue, especially in the most commonly affected age group, and therefore the cumulative space-occupying effect of ICH may not always be easy to define. Nevertheless, this interaction between cumulative space-occupying effect and brain tissue volume is probably crucial for critical intracranial and perihemorrhagic pressure elevation and therefore neurological status and outcome.

We tried to find a fast and simple method for assessing the combined mass effect of the hemorrhage and the edema with an indirect approach by analyzing the sulcal subarachnoid space. There are literally dozens of prognostic ICH outcome scores described in the literature (Ji et al., 2013; Schmidt et al., 2018) and the aim of this study was not to outperform any already existing score by reassessing established clinical and radiological parameters. The aim of this study was to provide a simple score assessing the combined mass effect of the hemorrhage and surrounding edema. Sulcal effacement subsumes numerous characteristics of an ICH, namely ICH volume, perihematomal edema, intraventricular volume and also a varying amount of ‘baseline’ brain tissue. Presence or absence of the subarachnoid space is easily and readily recognized on CT and was shown to be useful after subarachnoid hemorrhage (Ahn et al., 2018). Here we applied the principle of fast assessment of the subarachnoid space on CT imaging in patients with intracranial hemorrhage to propose a simple scoring system – the intracerebral mass and brain edema score (IMBES). We hypothesized that the resulting scoring system could predict early neurological outcome and mortality.

## 2 Material and methods

### 2.1 Selection of patients

Patients with spontaneous ICH treated at the Department of Neurosurgery of the Freiburg University Hospital between January 2009 and January 2019 were identified. Baseline clinical characteristics, radiological assessment of the ICH, the clinical course and treatment were retrospectively collected into an electronic database. Patient follow-up examination was included until 6 months after ICH. The primary outcome was the modified Rankin Scale (mRS) at discharge, key secondary outcome was the modified Rankin scale at early follow-up (66 days). Patients with a mRS of 4 or more are considered to be seriously disabled and not able to walk with or without assistance. The study was approved by the local institutional ethics committee (University of Freiburg, No. 131/19). Patient consent was neither required nor sought due to the retrospective data collection. This study has been registered as a clinical trial at the German Clinical Trials Register, German WHO primary registry (DRKS00034479). We included patients with spontaneous, non-traumatic, supratentorial ICH documented by CT scan, exclusion criteria were I) ICH due to a vascular malformation, ii) cerebellar or brainstem hemorrhage. All patients were treated according to institutional protocols and ICH guidelines (Steiner et al., 2014) including immediate reversal of anticoagulant therapy and control of the systolic blood pressure.

### 2.2 Radiological assessment

**Fig. 1:**
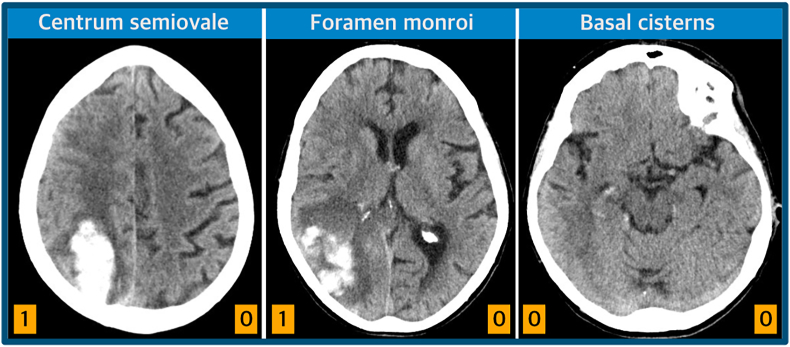
Intracerebral Mass and Brain Edema Score (IMBES). Three defined axial CT slices at the centrum semiovale, interventricular foramen of Monro and basal cisterns were visually inspected by two independent reviewers. Sulcal effacement of the subarachnoid space, involving more than half of the hemisphere, resulted in the assignment of one point. Assessment of the subarachnoid space by sulcal effacement for each hemisphere results in an IMBE-score between 0 and 6 points.

Acquired initial axial CT scans were assessed by two board certified independent reviewers, blinded for outcome, from the Department of Neuroradiology and Neurosurgery. ICH volume was calculated using abc/2 as described previously (Kothari et al., 1996). IMBES was calculated by visual inspection of three defined axial slices: at the level of (1) the centrum semiovale, (2) the interventricular foramen Monro and (3) the basal cisterns. CT slices were oriented parallel to the anterior commissure - posterior commissure line (AC-PC line), if feasible. Each side was assessed separately. If sulcal effacement of the subarachnoid space affected more than half of the hemisphere, one point was assigned. This resulted in an IMBE-score ranging from 0 to 6 points (Fig. 1).

After the CT scans were assessed by two blinded, independent reviewers, the intraclass correlation was performed for the single scores of the ICH volumetry and IMBES from each reviewer. The intraclass correlation coefficient (ICC) for the ICH volumes was 0.87 (95% CI: 0.84–0.89) showing an excellent correlation between the two reviewers (Koo and Li, 2016). This was also confirmed for the IMBES with an ICC of 0.84 (95% CI: 0.80–0.87). We calculated the mean of the measured ICH volumes from both reviewers. Discrepancies for the IMBES of more than 2 points between both reviewers were evaluated by a third, blinded external reviewer. The calculated mean values from the CT scoring of both reviewers were then rounded up if necessary.

### 2.3 Model comparisons

Established ICH scores were calculated as described in the original publications (Fahlstrom et al., 2020; Hemphill et al., 2001). However, since infratentorial ICH were excluded in this study, the calculated Hemphill-ICH score ranged from 0 to 5 points. Additionally, information regarding prior myocardial infarction was not available in this study, resulting in the Swedish-ICH score also ranging from 0 to 5 points. According to the Swedish-ICH score, the clinical-IMBES (cIMBES, 0–5 points) was defined as the sum of points assigned as follows: GCS score 15–13 (0 points), 12–5 (1 point), 4–3 (2 points); age ≥75 years (1 point); type 2 diabetes (1 points); IMBES<3 (0 points) IMBES ≥3 (1 point).

### 2.4 Statistical analysis

The main research hypothesis was that the IMBES could predict neurological outcome as measured by the primary outcome. All statistical analyses were performed using R (R version 4.3.1). All statistical tests were 2-sided and the level of significance was 0.05. Patients with missing information on mRS at discharge or CT volumetry were excluded from further statistical analysis. Inter-rater correlation coefficient was calculated using the R package *irr* and *psych*; the model was two-way, the type set to ‘agreement’, and the unit was ‘single’. Graphs were created using *ggplot2* in R. Correlation analysis was performed using Spearman's correlation. Distributions of the mean are based on the basic nonparametric bootstrap without assuming normality for confidence limits (*mean_cl_boot*). Univariate and multivariate ordinal regression were performed using the R package *ordinal*. Covariates for the multivariate model were selected using independent variables from the univariate model where the level of significance was below 0.25. Univariate logistic regression was calculated using *glm* in R with dependent variable setting to ‘binomial’. Multiple imputation was conducted using the R *mice* package to generate a comprehensive dataset for the subsequent multivariate logistic regression analysis. Stepwise logistic regression was then performed using the R function *step* based on Akaike's information criteria with 1000 steps to be considered. Odds ratios and 95% confidence intervals were calculated for covariates within the regression models. Residual deviance was then tested for goodness of fit using chi square cumulative distribution function and overdispersion using the dispersion test from the package *AER*. No underlying overdispersion was detected unless stated otherwise in the results. Receiver operating characteristic (ROC) curve analysis was conducted using the *pROC* and *plotROC* package in R. Akaike information criterion (AIC) from the R pacakage *AICcmodavg* was used to test the quality between the different models (ICH scores). The adjustment for small sample sizes is the corrected AIC (AICc).

## 3 Results

Within the mentioned time period 913 patients with spontaneous ICH were admitted to the Department of Neurosurgery. 140 patients were excluded due to cerebellar or brainstem hemorrhage. 11 patients were excluded due to missing clinical data. 762 patients were included in the final analysis (Table 1). Median age at admission was 75.4 years (IQR 64.3–81.1), 355 patients were female (46.6%). The mean length of stay for the acute inpatient treatment was 12.5 days (IQR: 7.1–22.3 days).

**Table 1:** Clinical baseline characteristics and radiological assessment.

Variable	Value
Age years, median (IQR)	75.4 (64.3–81.1)
Female sex, n/total	335/762 (46.6%)
Reported history of previous ICH, n/total (%)	64/762 (8.4%)
Reported history of previous stroke, n/total (%)	99/762 (13.0%)
Diagnosed diabetes mellitus, n/total (%)	124/762 (16.3%)
Diagnosed hypertension, n/total (%)	557/762 (73.1%)
Diagnosed renal insufficiency, n/total (%)	152/762 (19.9%)
Presence of coagulopathy, n/total (%)	46/762 (6.0%)
Intake of hyperlipidemia medication, n/total (%)	157/762 (20.6%)
Intake of antiplatelet therapy, n/total (%)	180/762 (23.6%)
Intake of anticoagulants, n/total (%)	254/762 (33.3%)
Initial systolic blood pressure, mean (SE)	156.5 mmHg (2.04)
Initial diastolic blood pressure, mean (SE)	81.2 mmHg (1.86)
Presence of bilateral pupillary light reflex, n/total (%)	641/762 (84.1%)
Intubated at admission, n/total (%)	314/762 (41.2%)
GCS at admission, median (IQR)	11 (3–14)
ICH volume, mean (SE)	46.1 cc (1.69)
Presence of IVH	458/762 (60.1%)
Location of ICH – deep	258/762 (33.8%)
Location of ICH – lobar	504/762 (66.1%)
Performance of any surgery (except EVD)	350/762 (45.9%)
Minimally invasive catheter drainage	255/350 (72.9%)
Craniotomy and clot removal	78/350 (22.3%)
Decompressive craniotomy with and without clot removal	17/350 (0.5%)
Value of IMBES assessed on initial CT	
0, n/total (%)	187/762 (24.5%)
1, n/total (%)	194/762 (25.5%)
2, n/total (%)	205/762 (26.9%)
3, n/total (%)	92/762 (12.1%)
4, n/total (%)	24/762 (3.1%)
5, n/total (%)	23/762 (3.0%)
6, n/total (%)	37/762 (4.9%)
Timepoint of discharge after admission: n/total (%), median (IQR)	560/762 (73.5%), 12.5 days (7.1–22.3)
Timepoint of control exam after admission: n/total (%), median (IQR)	293/762 (38.5%), 66.25 days (49.0–108.0)
Modified Rankin scale (mRS) at discharge	
0, n/total (%)	5/762 (0.7%)
1, n/total (%)	49/762 (6.4%)
2, n/total (%)	89/762 (11.7%)
3, n/total (%)	92/762 (12.1%)
4, n/total (%)	159/762 (20.9%)
5, n/total (%)	166/762 (21.8%)
6, n/total (%)	202/762 (26.5%)

### 3.1 Intracranial mass effect and brain edema score (IMBES) and neurological outcome

**Fig. 2:**
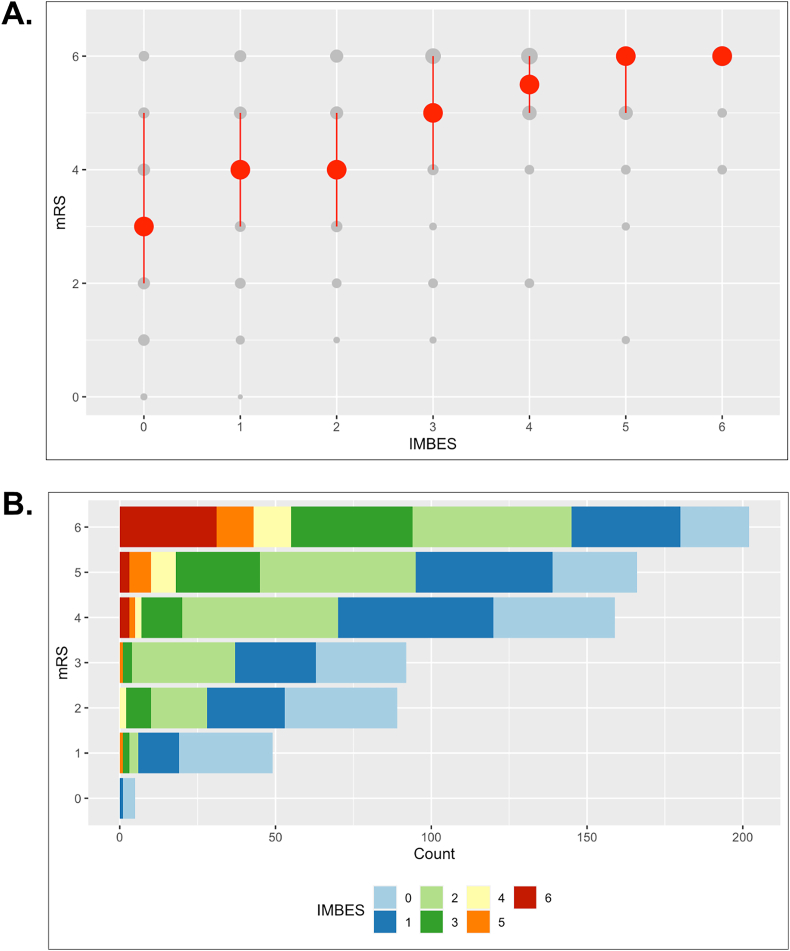
IMBES correlates with neurological outcome.**A.** Correlation of mRS at discharge and IMBES. Individual mRS values (0–6) are plotted in grey, the dot size represents the percentual proportion within the total count, red dots indicate medians, error bars represent 25%–75% point range quantiles. Spearman's correlation analysis confirmed a statistically significant correlation (p < 0.001). **B.** IMBES predicts detrimental neurological outcome. Colored bars represent the IMBE-score. High values on the IMBES (>3) are mostly present in patients with a detrimental neurological outcome at discharge (mRS >3).

Modified Rankin scale at discharge showed higher values with increasing IMBES (Fig. 2A). Patients with an IMBES of 0 points had a median mRS of 3 (IQR 2–5). Statistical analysis between mRS at discharge and the IMBES confirmed a robust correlation and association with worse outcome according to the mRS with increased IMBES (IMBES 1: mRS 4 [IQR 3–5], IMBES 2: mRS 4 [IQR 3–5], IMBES 3: mRS 5 [IQR 4–6], IMBES 4: mRS 5.5 [IQR 5–6], IMBES 5: mRS 6 [IQR 5–6], IMBES 6: mRS 6 [IQR 6-6], Spearman's correlation coefficient rho: 0.42, p < 0.001).

Moreover, IMBES greater than 3 were nearly exclusively present in patients with mRS of 4 and above (Fig. 2B), implying a prognostic value of the IMBES. Ordinal regression was performed for mRS at discharge as primary outcome. The baseline characteristics such as patients’ age, renal insufficiency, presence of coagulopathy, intake of antihypertensive drugs and anticoagulants revealed statistical significance for the univariate regression (Table 2). Surgical intervention after ICH also revealed statistical significance, which did not include the insertion of external ventricular drainage to monitor and lower intracranial pressure. ICH volume, the presence of intraventricular hemorrhage and the IMBES showed high statistical significance.

**Table 2:** Regression analysis for neurological outcome at discharge.

Univariate ordinal regression: mRS at discharge		
Characteristic	Odds Ratio (95% CI)	p-value
Age	1.02 (1.01–1.03)	<0.001 ∗∗∗
Gender (female)	1.14 (0.88–1.46)	0.32
Pre-existing hypertension	1.23 (0.87–1.75)	0.25
Any surgery (except EVD) performed	1.49 (1.16–1.93)	<0.01 ∗∗
ICH volume	1.02 (1.02–1.03)	<0.001 ∗∗∗
IMBES	1.81 (1.64–1.99)	<0.001 ∗∗∗
Presence of IVH	3.41 (2.61–4.47)	<0.001 ∗∗∗
Location of ICH (lobar)	0.88 (0.67–1.13)	0.31
Pre-existing diabetes mellitus	1.29 (0.91–1.83)	0.15
Pre-existing renal insufficiency	1.91 (1.38–2.67)	<0.001 ∗∗∗
Presence of coagulopathy	2.38 (1.38–4.14)	<0.01 ∗∗
History of previous ICH	1.34 (0.85–2.11)	0.21
History of previous stroke	0.97 (0.67–1.42)	0.89
Intake of antihypertensive drugs	1.38 (1.01–1.89)	<0.05 ∗
Intake of statins	1.38 (1.00–1.92)	0.054
Intake of anticoagulants	1.87 (1.39–2.51)	<0.001 ∗∗∗
Intake of antiplatelet therapy	1.06 (0.78–1.44)	0.68

Multivariate ordinal regression: mRS at discharge		
Characteristic	Odds Ratio (95% CI)	p-value
IMBES	1.44 (1.20–1.73)	<0.001 ∗∗∗
ICH volume	1.02 (1.01–1.03)	<0.001 ∗∗∗
Pre-existing renal insufficiency	2.13 (1.27–3.59)	<0.001 ∗∗∗
Age	1.03 (1.01–1.04)	<0.01 ∗∗
Presence of IVH	1.89 (1.27–2.81)	<0.01 ∗∗
Presence of coagulopathy	1.89 (0.92–3.92)	<0.05 ∗
Intake of anticoagulants	1.57 (1.01–2.46)	<0.05 ∗
Any surgery (except EVD) performed	0.63 (0.38–1.03)	0.07
History of previous ICH	1.82 (0.89–3.78)	0.10
Intake of antihypertensive drugs	0.63 (0.33–1.21)	0.16
Intake of statins	0.70 (0.44–1.10)	0.12
Pre-existing diabetes mellitus	1.38 (0.82–2.34)	0.22
Pre-existing hypertension	1.11 (0.56–2.21)	0.75

Multivariate ordinal regression was then performed including the independent variables from the univariate regression with statistical significance as covariates (p < 0.25). Here, age, ICH volume, presence of IVH, renal insufficiency, intake of anticoagulants and the IMBES accounted for statistical significance (Table 2).

### 3.2 Neurological outcome at early follow-up

Follow-up was available for 297 patients at a median of 66.7 days (IQR: 49.0–108.0 days). Increasing IMBES values showed ascending mRS values (Supplemental Fig. 1, Spearman's correlation coefficient rho: 0.28, p < 0.001). Median mRS at follow-up was 3 (IQR: 2–4; mRS 0: n = 15 (5.1%), mRS 1: n = 34 (11.4%), mRS 2: n = 61 (20.5%), mRS 3: n = 58 (19.5%), mRS 4: n = 73 (24.6%), mRS 5: n = 56 (18.9%)). Univariate ordinal regression with mRS at follow-up as the dependent variable was performed (Table 3). Adjusted multivariate regression confirmed statistical significance only for age, IMBES, history of previous stroke and location of ICH.

**Table 3:** Regression analysis for neurological outcome at control examination.

**Univariate ordinal regression: mRS at control examination**		
**Characteristic**	**Odds Ratios (95% CI)**	**p-value**
Age	1.02 (1.01–1.04)	<0.01 ∗∗∗
Gender (female)	1.05 (0.70–1.57)	0.81
Pre-existing hypertension	1.34 (0.81–2.20)	0.25
Any surgery (except EVD) performed	1.97 (1.31–2.97)	<0.01 ∗∗
ICH volume	1.02 (1.01–1.03)	<0.001 ∗∗∗
IMBES	1.53 (1.30–1.81)	<0.001 ∗∗∗
Presence of IVH	3.43 (2.24–5.28)	<0.001 ∗∗∗
Location of ICH (lobar)	0.46 (0.28–0.70)	<0.001 ∗∗∗
Pre-existing diabetes mellitus	1.31 (0.73–2.33)	0.36
Pre-existing renal insufficiency	1.08 (0.62–1.90)	0.78
Presence of coagulopathy	1.06 (0.46–2.46)	0.88
History of previous ICH	1.76 (0.70–4.57)	0.24
History of previous stroke	1.54 (0.85–2.78)	0.15
Intake of antihypertensive drugs	1.24 (0.77–1.99)	0.38
Intake of statins	1.29 (0.76–2.19)	0.35
Intake of anticoagulants	0.75 (0.47–1.20)	0.23
Intake of antiplatelet therapy	0.99 (0.60–1.62)	0.96
**Multivariate ordinal regression: mRS at control examination**		
Location of ICH (lobar)	0.22 (0.12–0.41)	<0.001 ∗∗∗
IMBES	1.37 (1.06–1.78)	<0.05 ∗
Age	1.03 (1.01–1.05)	<0.05 ∗
History of previous stroke	2.72 (1.26–6.00)	<0.05 ∗
ICH volume	1.01 (1.00–1.02)	0.07
Presence of IVH	1.62 (0.92–2.86)	0.10
Intake of anticoagulants	0.64 (0.37–1.10)	0.11
Any surgery (except EVD) performed	1.51 (0.76–3.01)	0.24
History of previous ICH	1.18 (0.39–3.72)	0.77

### 3.3 Mortality within the acute phase

202 of 762 patients died during acute care, resulting in an in-hospital mortality of 26.5%. In-hospital mortality was defined as the dependent variable for the univariate logistic regression analysis. The same covariates were selected as in the previous univariate regression. Statistical significance was revealed for multiple covariates (Supplemental Table 1). Subsequent multivariate stepwise logistic regression after multiple imputation of missing data was performed. Highest statistical significance was detected for IMBES (p < 0.001 ∗∗∗), age (p < 0.001 ∗∗∗) and performed surgery (p < 0.001 ∗∗∗).

### 3.4 Model validation and comparison

We performed a ROC analysis for a dichotomized neurological outcome (mRS 0–3 vs. mRS 4–6) and mortality within the acute phase. The area under the ROC curve indicated an acceptable discrimination for favorable vs. unfavorable outcome assessed by the IMBES (Supplemental Fig. 3). Furthermore, we tested for the quality of the clinical IMBE-score compared to already established ICH scores like the Hemphill ICH score (Hemphill et al., 2001) and Swedish ICH score (Fahlstrom et al., 2020). Among all considered scores, the cIMBES had the lowest AIC for neurological outcome at discharge and early follow-up, as well as mortality (Supplemental Table 2). The lowest AIC is generally considered as the best-fitting model and cIMBES indicated a slightly better performance compared to the Swedish-ICH. However, both cIMBES and Swedish-ICH indicated distinct differences from the Hemphill-ICH score.

## 4 Discussion

In this study the IMBES score based on sulcal effacement of the subarachnoid space on three axial CT planes was applied to the initial CT scans of 762 ICH patients. IMBES was the most robust parameter to predict neurological outcome and mortality at discharge as well as mRS at early follow-up. Although ICH volume is regarded as one of the major predictors of neurological outcome (Hemphill et al., 2001; LoPresti et al., 2014; Robinson et al., 2022) the IMBE-score outperforms ICH volume. ICH volume was not significantly associated with neurological outcome at the control examination. Reasons for this might be that the IMBES score not only assesses a single parameter like volume but also takes the edema volume into account as well as the individual subarachnoid anatomy. Based on the fact that the IMBES score assesses sulcal effacement, numerous characteristics of an ICH are included, namely ICH volume, perihematomal edema, intraventricular volume and also a varying amount of ‘baseline’ brain tissue. This allows a thorough assessment of the mass effect, which is not only exerted by the ICH volume alone. We defined this as ‘cumulative space-occupying effect’. In addition, IMBES is assessed on three easily defined and available axial CT-planes on imaging. This multilevel assessment might capture the extent of the ICH better than just the volume alone. The proposed three planes include sensitive anatomical structures like projection fibers of the corticospinal tract, thalamus and basal ganglia and internal and external capsules and last the basal structures including midbrain and brainstem.

Potentially IMBES may be used for prognostication during the early stages of clinical treatment following ICH onset. In the initial CT, IMBES primarily identifies the mass effect caused by ICH, with brain edema playing a more significant role in the space-occupying effect several days after ICH onset. Given that the ICH volume alone does not encompass the contribution of brain edema, IMBES has the potential to identify patients at risk of neurological impairment in subsequent CT imaging during the follow-up period.

### 4.1 Prognostic ICH scores

Various prognostic ICH scores are currently used within daily clinical practice and the Hemphill-ICH score being the most prominent (Hemphill et al., 2001; Mattishent et al., 2015). Modifications of this score have been described in the literature including pre-ICH cognitive impairment, NIHSS score, midline shift, ICH irregular shape, lobar location, hydrocephalus, hematoma enlargement and neurological deterioration (Mattishent et al., 2015; Masotti et al., 2021). A validation of the Hemphill-ICH-Score for 12-month functional outcome was reported in a prospective cohort predicting favorable 12-month outcome (mRS ≤4) for low values on the ICH score (Hemphill et al., 2009).

However, a prognostic tool to determine unfavorable neurological outcome is missing in daily clinical practice, since common ICH scores were validated for the prediction of favorable outcome. Besides the most commonly used Hemphill-ICH score at least 15 other scores were identified by recent literature research (Witsch et al., 2021). The scores are mainly used to differentiate between dichotomized endpoints (e.g. survival, favorable and unfavorable mRS categories) and vary in their complexity of included clinical information. Despite the high number of available ICH-scores only a minority is used in clinical routine which might be attributable to a reduced confidence in the prognostic tool or the perception that their use might not be time-efficient. The major advantage of the IMBES is that it is easy to assess, just by looking at the CT image and that no additional information or calculation is necessary. This makes its application in daily clinical practice possible. However, IMBES represents only a radiological score without the implication of replacing clinical scores but to add time-efficient radiological assessment into the decision making for the treatment of ICH patients.

As stated above, there are numerous scores to assess ICH outcome prognosis based on various clinical and radiological variables (Ji et al., 2013; Witsch et al., 2021). The Hemphill-ICH score is currently the most prominent score and attempts to establish new scores to better predict the neurological outcome have failed so far. External validation of the max-ICH score which includes the NIH stroke scale and the intake of oral anticoagulation as new variables has not demonstrated an improved prognostic performance (Schmidt et al., 2018). The aim of our study is to identify the combined impact of the mass effect of the hemorrhage and the edema rather than the bleeding volume alone and not to outperform existing scores.

### 4.2 Outcome and treatment of ICH

For decades, negative outcomes from major randomized controlled trials have fostered a pessimistic attitude within and outside the ICH community, resulting in a general perception that patients with ICH have a poor prognosis regardless of treatment (Seiffge and Anderson, 2024). However, recent studies have shown promising results in treating ICH. In 2023, randomized controlled trials demonstrated treatment benefits for a hyperacute care bundle approach (INTERACT3) (Ma et al., 2023), early minimally invasive hematoma evacuation (ENRICH)(Pradilla et al., 2024), and the use of factor Xa-inhibitor anticoagulation reversal with andexanet alfa (ANNEXa-I) (Connolly et al., 2024). These findings have been further reinforced by the confirmation that intensive blood pressure lowering initiated within the initial few hours of symptom onset can significantly improve outcomes in ICH (INTERACT4) (Chen et al., 2023) and that decompressive hemicraniectomy remains a viable treatment option for patients with large deep ICH (SWITCH) (Fischer et al., 2024). These findings might improve ICH care and patient outcomes (Seiffge and Anderson, 2024). The fast and simple identification of patients with space-occupying hemorrhages and edema, which otherwise would lead to a detrimental outcome, might be crucial for identifying the most accurate ICH treatment.

Therefore, we established a cutoff value for IMBES (IMBES<3 and IMBES ≥3) to determine patients at risk for impaired neurological outcome and mortality and combined the IMBES with clinical variables. This indicated a slight superiority to already established ICH scores like the Swedish-ICH or Hemphill-ICH score. The Swedish-ICH score has been introduced for a surgical patient cohort with supratentorial ICH. The Hemphill-ICH score has been primarily developed in conservatively treated ICH cohorts since only 13% of the patients included in the original study underwent surgical treatment.

Furthermore, we stratified patients based on the IMBES cutoff value (IMBES <3 and IMBES ≥3) and observed a clear advantage of surgical treatment for patients with IMBES ≥3 in terms of neurological outcomes at discharge and mortality within the acute care setting (data not shown). However, the established IMBES cutoff requires further investigation. While we observed excellent interrater reliability for the IMBES, real-world values may be less reliable due to the subjective nature of CT assessment for determining subarachnoid effacement.

### 4.3 Limitations

The results of the univariate and multivariate regression indicated statistical significance for several independent variables such as patients’ age, presence of IVH, renal insufficiency, intake of anticoagulants and location of ICH. Specifically, the presence of IVH has not been quantified in our study and the management after IVH has not been addressed when assessing the IMBES. Furthermore, there has been no specified limit for the timing of the initial CT scan. Potential progressive edema that may have developed after the initial ICH occurrence could have resulted in higher IMBES. These variables have to be taken into account when discussing the results of this study.

Potential selection bias may have occurred during patient selection. Only patients admitted to the Department of Neurosurgery have been included, which is reflected by a 66.1% prevalence of lobar ICH and a 45.9% prevalence of surgically treated patients. This may not reflect real-world conditions.

Furthermore, the study has the limitations of a retrospective analysis such as missing data, loss to follow-up, a posteriori defined outcome measures and changes in medical management that might introduce bias. The loss of follow-up at the latest time point was 47.7% compared to discharge from acute care. There might have been several reasons for this including cancellations of planned follow-up appointments, complications, or mortality.

Furthermore, other factors not included in the regression analysis may have influenced neurological outcomes and mortality rates. Although the study was conducted within a 10-year observation period at a single center, standard operating procedures have not been altered, thereby limiting the possibility of heterogeneity in medical treatment. In addition, shortcomings of the most commonly used formula (abc/2) for assessing ICH volume have been described and the use of other methods has been discussed (Zhao et al., 2020).

## 5 Conclusion

The proposed IMBE-score shows a strong independent association with outcome at discharge and two-month follow-up. IMBES combines the effect of hemorrhage and edema volume to assess the cumulative space-occupying effect, even outperforming ICH volume and other established parameters like age. This time-efficient assessment of the initial CT scan in ICH patients can be used to identify patients at high risk for impaired neurological outcome and provides evidence for daily clinical decision making in terms of expected neurological disability and mortality. It should be worthwhile to test IMBES prospectively.

## Human ethics and consent to participate declarations

The study was conducted according to the guidelines of the Declaration of Helsinki, and approved by the Institutional Ethics Committee of the Medical Center University of Freiburg (No. 131/19). According to the guidelines of the Institutional Ethics Committee, an additional consent to participate declaration from each human was not necessary due to the retrospective data collection.

## Authors’ contributions

Conceptualization: R.W., C.F., J.B.; methodology: R.W.; data acquisition: R.W., A.B., A.E., P.F., R.A.; data validation: R.W., A.B.; manuscript draft and graphical visualization: R.W., manuscript review and editing: R.W., P.S., A.E., P.F., A.B., M.H., R.R., M.S., O.S., S.E., C.T., C.F., J.B.; continuous supervision: R.W., C.F., J.B. All authors have read and agreed to the published version of the manuscript.

## Conflicts of interest and funding

The authors declare no conflict of interest. This study received no funding.

## Availability of data and material

The used dataset and details of the statistical analysis will be made available from the corresponding author on reasonable request.
