# Supplementary material for: A simple CT score predicts early neurological disability and survival in supratentorial intracerebral hemorrhage - The intracerebral mass and brain edema score (IMBES)
Source: Brain Spine. 2025 Feb 28;5:104226. doi: 10.1016/j.bas.2025.104226 (PMC11964648; doi:10.1016/j.bas.2025.104226)
Supplement: Multimedia component 1 [file mmc1.docx]

**Supplemental Table 1: Logistic regression for mortality during acute care**

| **Univariate logistic regression: mortality within acute phase** | | |
| --- | --- | --- |
| **Characteristic** | **Odds Ratios (95% CI)** | **p-value** |
| Age | 1.03 (1.01 – 1.04) | <0.001 *** |
| Gender (female) | 1.14 (0.82 – 1.58) | 0.44 |
| Pre-existing hypertension | 1.28 (0.86 – 1.89) | 0.22 |
| Any surgery (except EVD) performed | 0.82 (0.59 – 1.14) | 0.24 |
| ICH volume | 1.02 (1.02 – 1.02) | <0.001 *** |
| IMBES | 1.72 (1.54 – 1.94) | <0.001 *** |
| Presence of IVH | 3.14 (2.16 – 4.63) | <0.001 *** |
| Location of ICH (lobar) | 1.43 (1.01 – 2.06) | 0.047 * |
| Pre-existing diabetes mellitus | 1.21 (0.76 – 1.89) | 0.41 |
| Pre-existing renal insufficiency | 2.12 (1.41 – 3.15) | <0.001 *** |
| Presence of coagulopathy | 2.55 (1.35 – 4.70) | <0.01 ** |
| History of previous ICH | 1.34 (0.74 – 2.36) | 0.32 |
| History of previous stroke | 1.06 (0.62 – 1.73) | 0.84 |
| Intake of antihypertensive drugs | 1.58 (1.01 – 2.56) | 0.053 |
| Intake of statins | 1.42 (0.92 – 2.19) | 0.11 |
| Intake of anticoagulants | 2.19 (1.51 – 3.18) | <0.001 *** |
| Intake of antiplatelet therapy | 1.33 (0.91 – 1.92) | 0.13 |
| **Stepwise logistic regression: mortality within acute phase** | | |
| **Characteristic** | **Odds Ratios (95% CI)** | **p-value** |
| IMBES | 1.68 (1.42 – 2.00) | <0.001 *** |
| Age | 1.04 (1.02 – 1.06) | <0.001 *** |
| Any surgery (except EVD) performed | 0.35 (0.21 – 0.55) | <0.001 *** |
| Presence of IVH | 1.86 (1.21 – 2.88) | <0.01 ** |
| ICH volume | 1.01 (1.00 – 1.02) | <0.01 ** |
| Pre-existing renal insufficiency | 2.11 (1.37 – 3.24) | <0.001 *** |
| Presence of coagulopathy | 4.03 (2.08 – 7.88) | <0.001 *** |
| History of previous ICH | 1.96 (1.09 – 3.47) | <0.05 * |

**Supplemental Table 1**: Logistic regression for mortality during acute care. Univariate logistic regression was performed with death within the acute phase as the dependent variable. Stepwise logistic regression was then performed after multiple imputation of missing dataet using all variables from the univariate logistic regression. Odds ratio, 95% confidence intervals (CI), and p-values are shown. Asterisks indicate statistical significance: * p<0.05, **p<0.01, ***p<0.001.

**Supplemental Table 2: Akaike information criterion (AIC) analysis**

| **mRs at discharge** | **AICc** | **Delta** |
| --- | --- | --- |
| cIMBES | 1948.70 | 0.00 |
| Swedish-ICH | 1965.85 | 17.15 |
| Hemphill-ICH | 2249.98 | 301.28 |
| **mRs at follow-up** | **AICc** | **Delta** |
| cIMBES | 794.94 | 0.00 |
| Swedish-ICH | 799.63 | 4.69 |
| Hemphill-ICH | 895.70 | 100.76 |
| **Mortality within acute care** | **AICc** | **Delta** |
| cIMBES | 566.50 | 0.00 |
| Swedish-ICH | 573.07 | 6.57 |
| Hemphill-ICH | 687.60 | 121.10 |

**Akaike information criterion (AIC) analysis for best-fitting model.** cIMBES had the lowest AICc for neurological outcome at discharge and early follow-up, as well as mortality among all considered scores. The corrected AIC is an adjusted AIC for small samples and the lowest AICc is considered the best-fitting model. cIMBES slightly outperformed the Swedish-ICH, but both differed from the Hemphill-ICH score.

**Supplemental Figure 1**


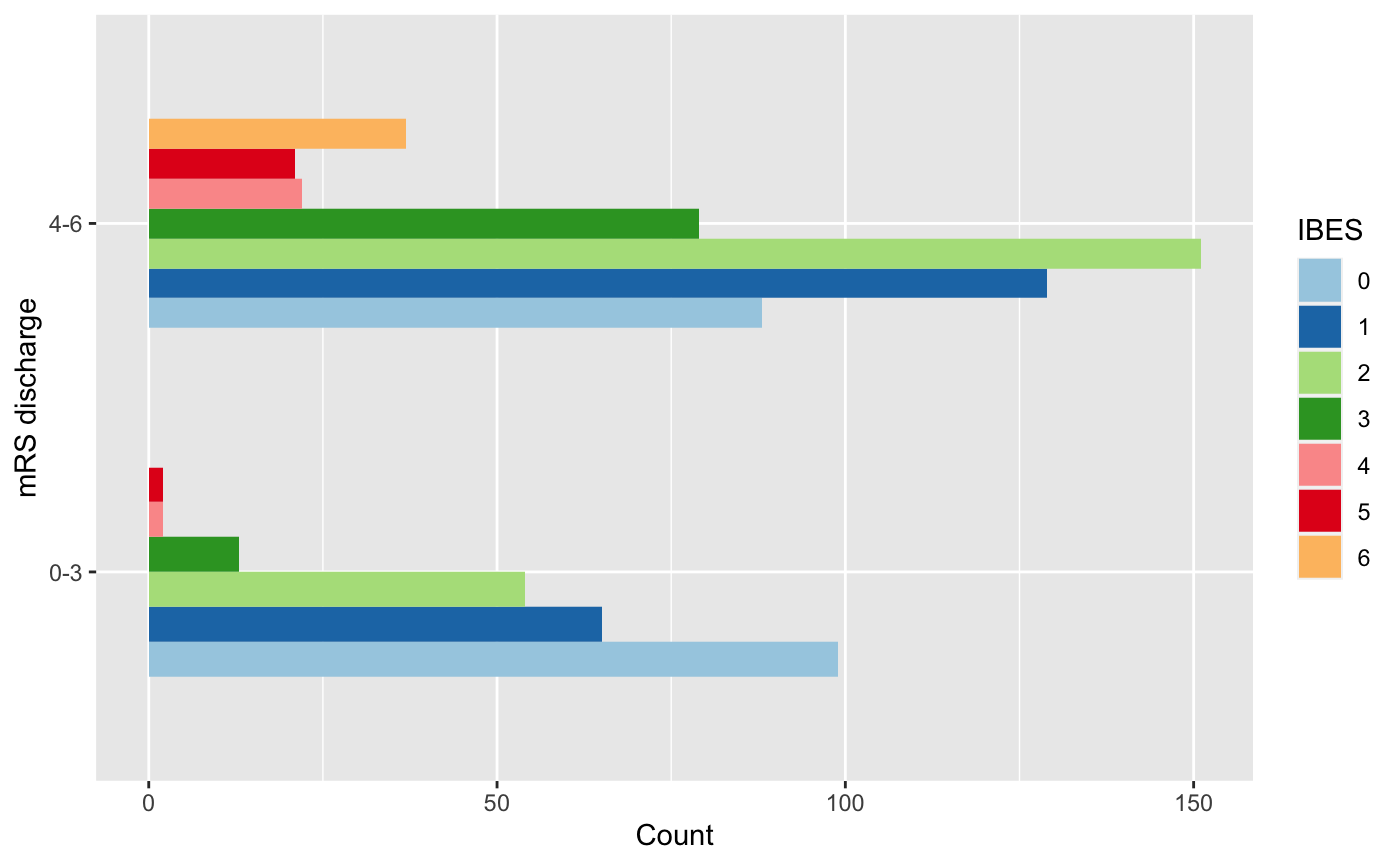


**IMBES correlates with mRS at discharge**. Colored bars represent the acquired rating on the IMBES. High values on the IMBES (>3) are mostly present in patients with detrimental neurological outcome at discharge (mRS >3).

**Supplemental Figure 2**


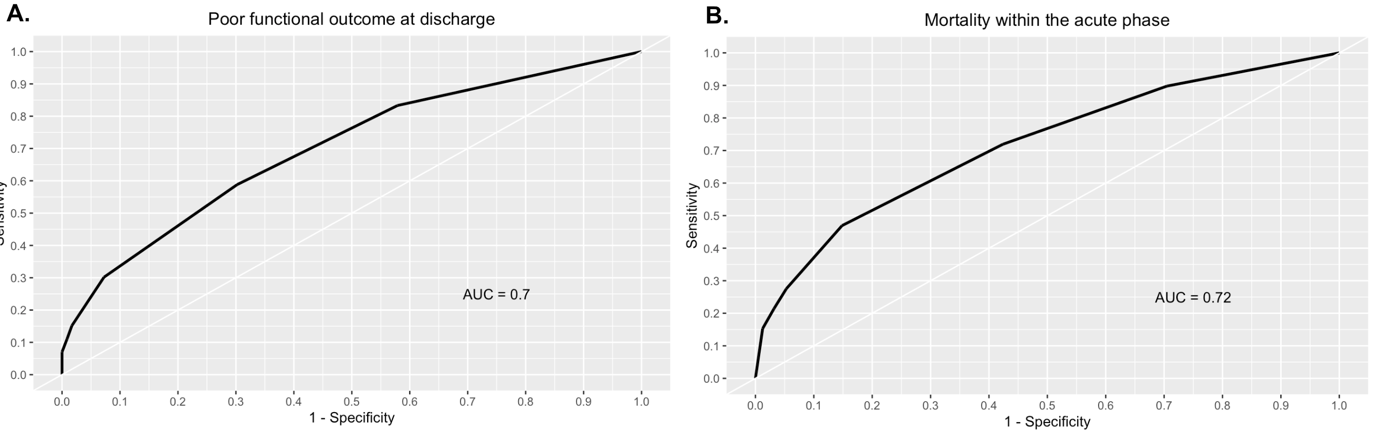


**Receiver operating characteristic curve analysis**. **A.** ROC analysis for good (mRS 0-3) vs poor (mRS 4-6) outcome, AUC 0.70 (95% CI: 0.66 – 0.74) **B.** ROC analaysis for mortality within the acute phase, AUC 0.72 (95% CI: 0.67 – 0.76)

**Supplemental Figure 3**

**
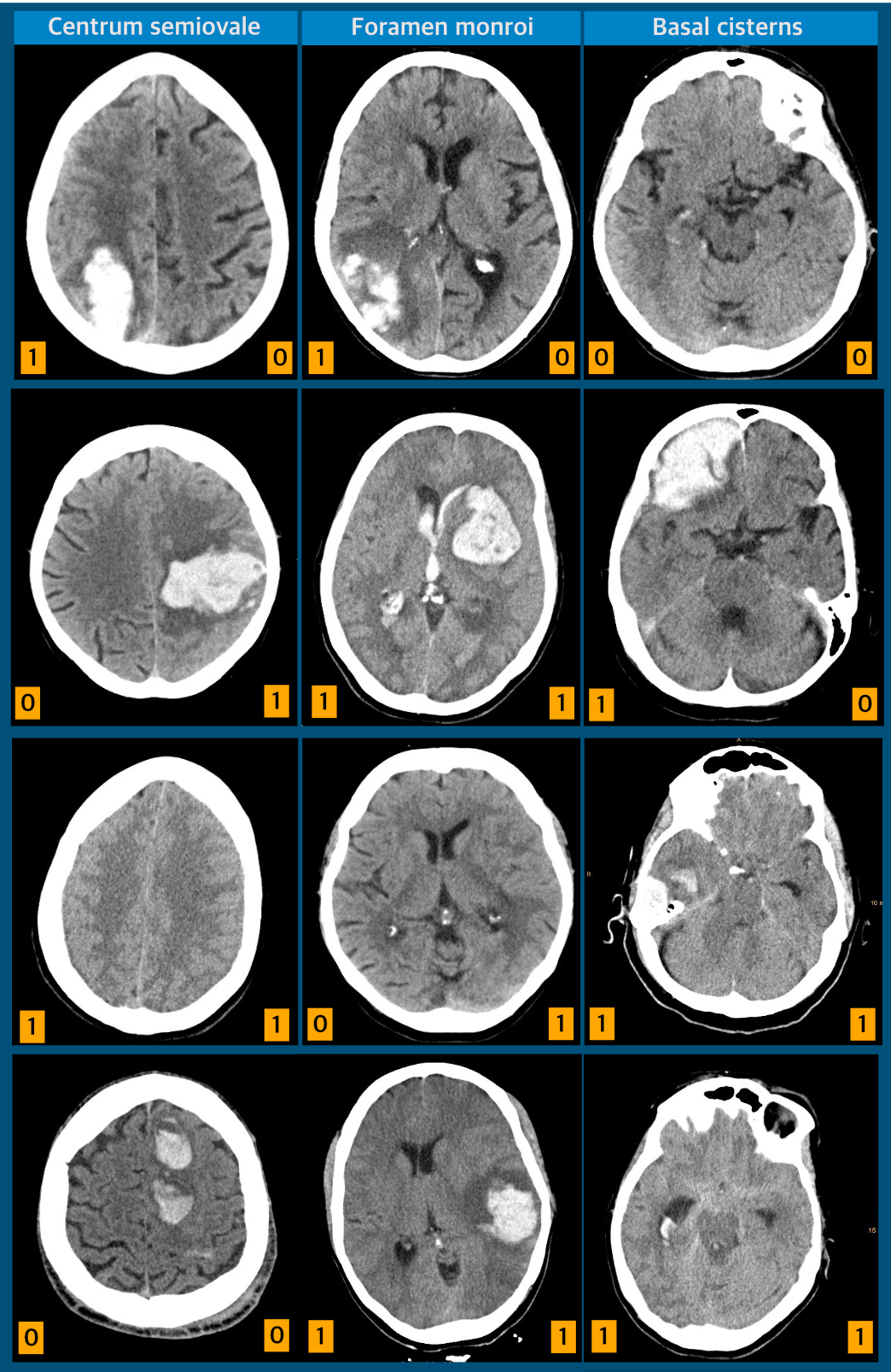
**

**Intracerebral Mass and Brain Edema Score (IMBES).** Four examples of IMBES scoring are shown here. The first row shows the identical CT slides from Figure 1 and row 2-4 represent additional exemplary CT scorings using the IMBES. The sum of these three different axial planes results in an IMBES from 0-6 points for each patient.
